# Compliance-by-Design in an AI-Enabled Platform for Early Crohn Disease Interception: A Viewpoint

**DOI:** 10.2196/94570

**Published:** 2026-11-09

**Authors:** Franco Scaldaferri, Pierluigi Puca, Elena Fiorelli, Judith Bosmans, Richard Vesely, Geert D'Haens, Jean-Frederic Colombel, Joana Torres, Antonio Gasbarrini, Alfredo Cesario

**Affiliations:** 1 Centro per le Malattie Apparato Digerente (CEMAD), IBD Unit, UOC Medicina Interna e Gastroenterologia, Agostino Gemelli University Polyclinic, Rome, Lazio, Italy; 2 Gemelli Digital Medicine and Health S.r.l, Largo Agostino Gemelli 8, Rome, Lazio, 00168, Italy, 39 3382040515; 3 Department of Health Sciences, Faculty of Science, Vrije Universiteit Amsterdam, Amsterdam, The Netherlands; 4 R Consulting, Laren, The Netherlands; 5 Department of Gastroenterology and Hepatology, Amsterdam University Medical Centers, Amasterdam, North Holland, The Netherlands; 6 Icahn School of Medicine at Mount Sinai, New York, NY, United States; 7 Division of Gastroenterology, Faculdade de Medicina, Hospital Beatriz Ângelo, Lisbon, Portugal; 8 Unità di Medicina Interna e Gastroenterologia, Dipartimento di Medicina e Chirurgia Traslazionale, Agostino Gemelli University Polyclinic, Rome, Lazio, Italy

**Keywords:** artificial intelligence, AI, compliance-by-design, Crohn disease, digital medicine, federated learning, medical device software, regulatory science

## Abstract

AI-enabled digital medicine platforms in Europe are developed within overlapping rules for personal data, medical devices, AI, clinical research, health data access, and health technology assessment. This viewpoint represents the authors’ experience and interpretation from the preimplementation phase of INTERCEPT, an Innovative Health Initiative consortium that plans to validate biomarkers and develop a blood-based risk score for early Crohn disease interception. It does not report original empirical research, a systematic regulatory review, a deployed platform, or a validated AI model. Our aim is to explain how compliance-by-design can be translated into an auditable governance architecture and to identify what still requires scientific evaluation. We advance 3 messages. First, governance must begin with intended purpose and boundary mapping: a digital platform may contain research, operational, and medical device software functions, and conformity of one module cannot be inferred for the platform as a whole. Second, overlapping obligations can be operationalized through 5 linked controls: modular responsibility and change control, federated data governance, traceable AI life cycle management, consent and participant rights orchestration, and a common cross-border governance core with local adaptations. Third, compliance is not a surrogate for model validity, clinical benefit, trust, scalability, or market access. Before clinical deployment, planned AI components require prespecified labels and cohorts, leakage prevention, missing data and imbalance strategies, calibration, subgroup assessment, external validation, human factors evaluation, and postdeployment algorithmovigilance. A hypothetical biomarker risk score update illustrates how the 5 controls interact. INTERCEPT is informative because it combines cross-border biomarker research, planned predictive analytics, and heterogeneous institutional settings while key design decisions remain modifiable. Its contribution is nevertheless conceptual: the proposed framework is a testable governance hypothesis, not evidence of implementation success. Prospective studies should compare it with alternative governance models and assess safety, workload, participant experience, evidence generation, and regulatory performance.

## Introduction

Digital medicine increasingly combines software, distributed data infrastructures, biomarkers, and AI-enabled analytics. A systematic review of registered digital therapeutic trials documented substantial growth and wide variation in intervention types, outcomes, and evidentiary maturity, underscoring that technical innovation and clinical evidence do not advance at the same pace [1].

INTERCEPT is a 5-year Innovative Health Initiative project launched in 2025. Public project information describes a consortium of 21 partners from 16 countries that aims to discover and validate biomarkers and develop a blood-based risk score to identify people at increased risk of Crohn disease before clinical onset [2]. The project therefore sits at the intersection of biomarker research, multisite data governance, planned predictive modeling, and future clinical evaluation.

In Europe, this type of development takes place across partially overlapping legal and policy regimes, including the General Data Protection Regulation (GDPR), Medical Device Regulation (MDR), In Vitro Diagnostic Medical Devices Regulation (IVDR), AI Act, European Health Data Space (EHDS), Clinical Trials Regulation (CTR), and health technology assessment (HTA) Regulation [3-9].

This article is deliberately framed as a viewpoint rather than an empirical study. We argue that compliance-by-design should be treated as an architecture and governance discipline established before model development and deployment. Our aim is to make that proposition operational through 3 messages: platform-level governance must be separated from module-level conformity assessment, 5 linked design controls can translate overlapping requirements into decisions and evidence, and regulatory alignment must remain distinct from scientific validation and demonstrated implementation benefit.

## Basis, Scope, and Aim of This Viewpoint

The viewpoint is informed by the authors’ multidisciplinary participation in the early planning of INTERCEPT and by a targeted reading of publicly available European legislation, official guidance, national pathway documents, and peer-reviewed literature. It is not a systematic or scoping review. No eligibility protocol, stakeholder survey, qualitative coding exercise, or formal consensus method was used to derive the 5 controls. They are an interpretive framework for organizing recurrent planning questions rather than empirically derived constructs.

No individual-level data, patient outcomes, or confidential consortium deliverables are analyzed in this article. The hypothetical use case is illustrative and does not report an implemented workflow. Because the viewpoint contains no human participant research or original data collection, research ethics approval was not applicable. This scope also means that the article should not be read as legal advice, a conformity assessment, or evidence that the proposed architecture is feasible or superior.

## Why INTERCEPT Is an Informative Preimplementation Case

INTERCEPT is informative not because it has demonstrated a successful digital platform, but because several regulatory boundaries must be considered while design choices are still reversible. The planned program links biomarker discovery and validation, a blood-based risk score, cross-institutional data use, and a future prevention-oriented clinical pathway [2]. These elements can fall under different governance regimes depending on intended purpose, users, claims, and stage of development.

The multinational consortium also exposes a practical distinction that can be obscured in single-site projects: European regulations may be directly applicable, yet operational requirements still vary through ethics procedures, institutional infrastructure, national reimbursement pathways, and local interpretations. A preimplementation case therefore permits examination of governance choices before they are embedded in code, contracts, data flows, and clinical routines. The trade-off is that no claim of real-world performance can yet be made.

## A Layered Regulatory Problem, Not a Single Checklist

Terminology is consequential in regulatory writing. In this viewpoint, a digital health platform is the broader technical and organizational infrastructure and is not assumed to be a medical device. Digital medicine refers to evidence-generating digital technologies used to measure, understand, or intervene in health. Digital therapeutics are a narrower category of software-based therapeutic interventions and are discussed only when describing national access pathways. Medical device software is software with an intended medical purpose that meets the applicable MDR or IVDR definition. An AI-enabled component is a planned analytical function; the label does not determine whether the surrounding platform is itself a regulated product.

Table 1 translates the principal European frameworks into developer-facing implications. The table does not imply that every framework applies to every INTERCEPT activity. Applicability depends on the specific processing operation, intended purpose, product claim, study design, and deployment context.

**Table 1.** Principal European frameworks and concrete implications for developers of AI-enabled digital medicine systems.

Framework	Core relevance	Concrete developer implications	Key interface, uncertainty, or timing issue
GDPR^a^; Regulation (EU^b^) 2016/679 [3]	Lawful and fair processing of personal data, Article 25 data protection by design and default, and security and enforceable rights	Maintain data-flow and role maps; document lawful basis and Article 9 condition; perform a data protection impact assessment where required; and implement minimization, access, retention, security, and rights workflows.	Research consent and data protection legal bases are distinct. Representative subgroup evaluation may require protected attributes. Federation does not by itself remove personal data status.
MDR^c^ and IVDR^d^; Regulations (EU) 2017/745 and 2017/746 [4,5]	Qualification and classification by intended purpose; quality management; risk, clinical, or performance evaluation; cybersecurity; and postmarket obligations	Map product and module boundaries; identify manufacturer and dependencies; classify MDSW^e^ or IVD functions; and plan evidence, usability, cybersecurity, release control, postmarket surveillance, and update assessment.	Modularity does not automatically permit separate certification. A software, assay, or interface change may alter intended purpose, risk, or system-level evidence [10].
AI Act; Regulation (EU) 2024/1689 [6]	For high-risk systems when applicable: risk and data governance, technical documentation, logs, human oversight, accuracy, robustness, cybersecurity, and monitoring	Align AI risk files and logs with MDR or IVDR quality processes and define data representativeness, oversight, performance, incident, and change controls before deployment.	Application is phased, and implementing guidance is evolving. AI Act and device obligations can be coordinated but are not identical [11,12].
EHDS^f^; Regulation (EU) 2025/327 [7]	Rules and infrastructure for primary and secondary use of electronic health data, secure processing, interoperability, and data quality	Prepare metadata, provenance, interoperability, data quality, permit, secure processing, and output-control processes that can operate with national health data access bodies.	Implementation is staged and national infrastructure will mature unevenly. EHDS does not displace GDPR, research ethics, or local governance.
CTR^g^; Regulation (EU) 536/2014 [8]	Authorization, conduct, safety reporting, informed consent, and records for medicinal product clinical trials through the Clinical Trials Information System	Where an interception trial includes a medicinal product, align digital end points and systems with protocol, validation, retention, safety, and change requirements.	The boundary among a research platform, a medical device investigation, an IVD^h^ performance study, and a medicinal product trial must be mapped explicitly.
HTA^i^ Regulation; Regulation (EU) 2021/2282 [9]	Joint clinical assessment and scientific consultation for covered technologies, alongside national appraisal and reimbursement decisions	Plan comparative evidence beyond conformity assessment: patient-relevant outcomes, comparator, workflow impact, resource use, economic evidence, and budget consequences.	Digital-specific methods and coverage pathways remain heterogeneous; CE marking does not establish value or reimbursement.

a GDPR: General Data Protection Regulation.

b EU: European Union.

c MDR: Medical Device Regulation.

d IVDR: In Vitro Diagnostic Medical Devices Regulation.

e MDSW: medical device software.

f EHDS: European Health Data Space.

g CTR: Clinical Trials Regulation.

h IVD: in vitro device.

i HTA: health technology assessment.

## Where the Frameworks Align—and Where Friction Remains

The frameworks share a life cycle orientation but are not interchangeable. GDPR asks whether personal data processing has a lawful basis, an Article 9 condition, proportionate safeguards, and enforceable data-subject rights [3]. MDR and IVDR ask whether software or diagnostic functions have a medical purpose and whether safety, performance, clinical evidence, quality management, and postmarket obligations are satisfied [4,5,10]. The AI Act adds risk management, data governance, documentation, logging, human oversight, accuracy, robustness, and cybersecurity duties when its high-risk provisions apply [6,11,12]. EHDS may facilitate defined primary and secondary uses of health data, but it does not displace GDPR or local ethical governance [7]. HTA and national reimbursement bodies may require comparative, organizational, and economic evidence that is not established by CE marking [9].

Several tensions require explicit design decisions. Data minimization can conflict with the need for sufficiently representative data and protected attributes to test subgroup performance. Federated analysis can reduce transfers of raw records but does not automatically anonymize inputs, eliminate reidentification or model-inference risks, or resolve inconsistent local data quality. MDR and IVDR and AI Act quality systems may be coordinated, but their scopes, terminology, and transition timelines are not identical [11,12]. EHDS implementation is staged, so developers must design for future interoperability without assuming that data access, permits, and infrastructure are already uniform across member states.

Legal compliance, ethical acceptability, and implementation readiness are therefore related but distinct. Legal compliance is the minimum conformance required by applicable law. Ethical acceptability asks whether a lawful design is proportionate, understandable, fair, and responsive to participants and affected groups. Implementation readiness asks whether evidence, workflow integration, usability, cybersecurity, accountability, financing, and operational capacity support safe routine use. Passing one test does not establish the other two.

## National Implementation Adds a Second Layer of Heterogeneity

European product rules coexist with national access and reimbursement arrangements. Table 2 provides 4 current illustrations rather than a validated classification of all member states. These pathways primarily concern digital medical devices or digital therapeutics; they should not be applied automatically to a research platform or a biomarker risk model. Their value here is to show that a product may face different evidence sequencing, coverage mechanisms, and institutional interfaces after conformity assessment.

**Table 2.** Illustrative national access and reimbursement pathways for digital medical products.

Country and pathway	Illustrative feature	Implication for planning	Boundary of this example
Germany—DiGA^a^ Fast-Track [13]	BfArM^b^ assesses eligible lower-risk digital health applications for listing in a reimbursable directory, and provisional listing can support evidence generation.	Product scope, data protection, interoperability, usability, and the study plan for positive health care effects must be designed early.	This pathway applies to eligible digital medical devices, not to every digital platform, AI model, or diagnostic function.
France—PECAN^c^ [14]	One year of exceptional coverage can be available for sufficiently mature therapeutic digital medical devices or remote monitoring activities while evidence is completed.	The transition from early coverage to ordinary reimbursement requires a defined evidence and submission pathway.	This pathway requires an eligible, CE-marked digital medical device and should not be generalized to research infrastructure.
Belgium—mHealthBelgium validation pyramid [15]	A 3-level framework links regulatory, technical, clinical, and socioeconomic maturity to higher levels and potential reimbursement.	Evidence and interoperability requirements are staged and involve several national actors.	The pyramid is a national validation and access mechanism, not a European Union–wide classification scheme.
Italy—digital therapeutics bill [16]	A unified bill on digital therapeutics was approved by the Chamber of Deputies in May 2026 and transmitted to the Senate.	The proposal signals movement toward dedicated evaluation and governance, but operational requirements depend on the final law and implementing measures.	As of June 23, 2026, the proposal had not completed the parliamentary process and did not create a current reimbursement entitlement.

a DiGA: Digitale Gesundheitsanwendungen (digital health applications).

b BfArM: Federal Institute for Drugs and Medical Devices.

c PECAN: prise en charge anticipée numérique (advance digital care).

## Five Compliance-by-Design Controls

### Intended Purpose Mapping, Modular Responsibility, and Change Control

The first control is a maintained map of functions, intended users, inputs, outputs, clinical claims, legal roles, and dependencies. “The platform” is too coarse a unit for regulatory analysis. A research data workspace, an assay, a clinician-facing risk score, and an operational dashboard may require different evidence and accountability even when they share infrastructure.

Modularity can make responsibilities and updates more manageable, but it does not create automatic entitlement to separate certification. Medical Device Coordination Group guidance emphasizes intended purpose and the independent function of software modules; interfaces and collective functionality must also be considered [10]. For each module, the governance record should identify the responsible organization, manufacturer status where relevant, data controller or processor roles, applicable quality system, cybersecurity dependencies, validation evidence, release authority, and change owner. An algorithm update, new input variable, changed threshold, or altered user interface should trigger a documented assessment of effects on intended purpose, performance, risk controls, and regulatory status. Where dependencies are safety critical, system-level testing remains necessary even if components are managed separately.

### Federated Data Governance and Privacy Engineering

The second control is an explicit federation design rather than a general promise that “data remain local.” Federated analysis can send approved code to institutional data and return aggregates, statistics, or model parameters. DataSHIELD and the MOLGENIS Armadillo server illustrate infrastructure for remote analysis without routine transfer of individual-level data [17,18]. Differential privacy, secure aggregation, and related techniques can further reduce some disclosure risks, although they introduce accuracy, complexity, and governance trade-offs [19]. These methods are cited as design options; this viewpoint does not claim that INTERCEPT has implemented a particular privacy-enhancing technology.

A credible federation plan must address nonidentical data distributions, assay and coding differences, missing variables, small sites, latency, outages, software-version drift, and local compute capacity. It must also consider membership-inference, model-inversion, and malicious-update threats. Candidate controls include a common data model with local mapping reports; prespecified data quality checks; minimum-cell and disclosure rules; encrypted transport; role-based query approval; reproducible analysis containers; site-level validation; and a record of every query, code version, output, and disclosure decision. Federation changes the location of risk; it does not remove the need for governance.

### A Traceable AI Life Cycle

The third control is a traceable life cycle for any future AI component. INTERCEPT has not yet produced a scientifically validated model for clinical use. Public project descriptions envisage a blood-based risk score, but the final input modalities, target label, algorithm class, feature engineering, training strategy, and clinical thresholds are not specified in this viewpoint [2]. Language suggesting model readiness has therefore been avoided.

Before development begins, the technical and regulatory record should define dataset and label versions, permissible preprocessing, cohort partitions, software environments, performance objectives, human oversight, and release criteria. During development, traceability can be supported through version-controlled code; immutable dataset snapshots or hashes; model registries; structured experiment records; access logs; approval signatures; and documented links among requirements, tests, risks, and releases. Deployment should use a named version, monitored interfaces, a rollback path, and a prohibition on uncontrolled self-learning. These controls support auditability, but they do not establish scientific validity by themselves [6,11,12]. They also create the documentation base for explainability, usability, and transparency, which are central to trustworthy clinical AI [20].

### Consent and Participant Rights Orchestration

The fourth control is a rights and permissions layer that distinguishes research informed consent from the legal bases used for personal data processing. GDPR consent is not the only possible lawful basis, and withdrawing study participation does not necessarily erase every record that must be retained for safety, scientific integrity, or legal obligations. Treating all permissions as one binary switch can therefore mislead participants and create inconsistent system behavior.

A layered approach can record separate choices for core study participation, optional secondary analyses, biospecimen reuse, recontact, and data sharing, while a clear interface explains consequences [21]. For partial withdrawal, the system should stop future optional processing prospectively, propagate the updated status to affected sites and analysis queues, preserve an auditable history, and identify data or results that cannot lawfully or practically be retracted. A common European permissions model can provide the core categories, with country- or site-specific annexes where ethics or legal requirements differ. Conflicts should be escalated to a designated governance body rather than resolved silently in software.

### A Cross-Border Governance Core With Local Overlays

The fifth control is a shared governance core that can accommodate legitimate local variation. A practical core may include a responsibility matrix, data-processing and joint-controller arrangements where applicable, a data protection impact assessment template, common data definitions, an evidence matrix, security and incident procedures, change-control rules, audit schedules, and decision rights for releases and suspensions.

Adopting the strictest rule encountered in any country as the universal default can appear simple but may be unnecessarily restrictive and still miss context-specific obligations. A more defensible model establishes a common floor based on directly applicable law and project-wide ethical commitments, then documents justified local overlays. The resulting deviations should be visible, versioned, and reviewed for their effects on data comparability, participant communications, timelines, and evidence. Harmonization is thus managed as configuration governance, not assumed as uniformity.

## Illustrative Use Case: Updating a Biomarker Risk Score Module

Figure 1 applies the 5 controls to a hypothetical decision to add a biomarker feature to a planned risk score module. First, the team confirms whether the output remains research stratification or becomes clinician-facing decision support; that intended purpose decision determines the relevant regulatory boundary and evidence. Second, each site verifies the permitted data use, assay comparability, missingness, and local data quality before any federated command is approved. Third, the development environment records the data dictionary, code, parameters, participating sites, privacy settings, and outputs for each run.

Fourth, the candidate version is locked and evaluated in temporally or geographically independent data that were excluded from training, with calibration, subgroup performance, and clinically meaningful thresholds prespecified. Fifth, any release is authorized for a named version, defined users, and a documented human-oversight workflow. Finally, algorithmovigilance [22] monitors performance, incidents, data drift, subgroup signals, and workflow consequences; predefined triggers can pause use, initiate investigation, or require a new conformity and validation assessment. Cross-border governance determines who can approve each transition and how a local deviation affects the consortium-wide release. The example is intentionally prospective and does not describe a validated INTERCEPT model.

**Figure 1.**
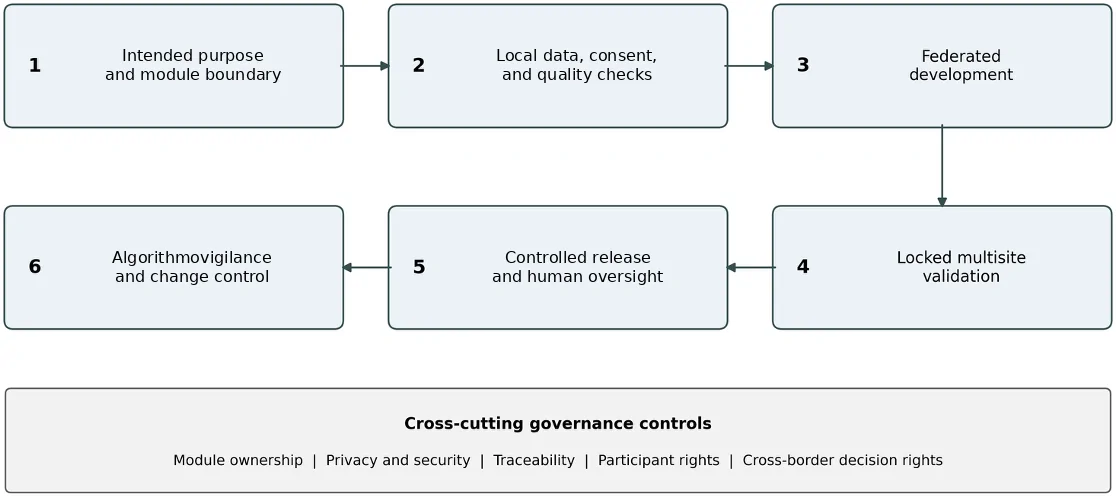
Hypothetical compliance-by-design workflow for a biomarker risk score update. The workflow links intended purpose, local rights and data quality checks, federated development, locked multisite validation, controlled release, and algorithmovigilance. It is a prospective example; no INTERCEPT model is currently validated or deployed.

## Scientific Evaluation Remains a Separate Obligation

Compliance-by-design can create the conditions for reproducible evaluation, but it cannot substitute for it. Table 3 converts the technical gaps identified by the reviewers into prospective requirements for any future model development protocol. These requirements are consistent with TRIPOD+AI (Transparent Reporting of a Multivariable Prediction Model for Individual Prognosis or Diagnosis–AI) reporting guidance, PROBAST+AI (Prediction Model Risk of Bias Assessment Tool for AI) risk-of-bias assessment, DECIDE-AI (Developmental and Exploratory Clinical Investigation of Decision-support systems driven by Artificial Intelligence) recommendations for early clinical evaluation, and the FUTURE-AI (Fairness, Universality, Traceability, Usability, Robustness, and Explainability in Artificial Intelligence) consensus guideline for trustworthy and deployable health AI [23-26].

The choice of algorithm should follow the intended use, data structure, sample size, missingness, interpretability needs, and deployment constraints rather than an assumption that greater complexity is preferable. Performance should be evaluated with uncertainty intervals and decision-relevant measures, not a single discrimination statistic. External validation must test transportability across sites, assays, populations, and time. Human factors and workflow evaluation are also required because a well-calibrated model can still cause harm through poor presentation, automation bias, alert burden, or unclear accountability.

**Table 3.** Prospective scientific safeguards required before clinical use of a planned AI risk score component.

Validity risk	Prospective safeguard	Evidence expected before clinical use
Unclear intended use or target label	Prespecify intended user, decision, time horizon, outcome definition, labeling process, and clinically meaningful action.	Protocol and analysis plan showing label reliability, outcome ascertainment, and alignment between model output and clinical pathway
Data leakage and overfitting	Partition by person and, where relevant, family, site, and time before preprocessing; keep validation data isolated; and document all tuning.	Reproducible code, locked model, audit of leakage pathways, optimism-corrected development estimates, and untouched validation results
Missingness, assay variation, and label noise	Characterize mechanisms and site-specific patterns and predefine imputation, measurement harmonization, quality thresholds, and sensitivity analyses.	Missingness tables, assay comparability results, sensitivity analyses, and performance under realistic incomplete-data scenarios
Class imbalance and spectrum bias	Use sampling and metrics appropriate to event prevalence, recruit the intended use spectrum, and avoid case-control estimates that are presented as deployment performance.	Precision-recall and decision metrics with CIs, prevalence-aware calibration, and subgroup results across the intended spectrum
Site effects and limited transportability	Quantify heterogeneity, use site-aware development, reserve external sites or later time periods, and evaluate federated and centralized-equivalent pipelines where feasible.	External validation across institutions, assays, populations, and time, with heterogeneity and failure analyses
Poor calibration or unjustified thresholds	Assess calibration-in-the-large, slope, curves, and decision consequences and prespecify threshold selection and recalibration rules.	Calibration plots and estimates, clinical utility or decision-curve analyses, and threshold-specific benefits and harms
Unequal performance or access	Predefine clinically relevant subgroups and fairness questions, ensure sufficient representation, and investigate data and workflow causes of disparities.	Subgroup performance with uncertainty, error analysis, mitigation assessment, and an equity monitoring plan
Human factors and workflow failure	Evaluate comprehension, automation bias, alert burden, override, escalation, and accountability with intended users in realistic workflows.	Usability and early clinical evaluation evidence, documented human oversight, and clearly assigned responsibility [25,26]
Drift, incident, or unsafe update after release	Set baselines, monitoring frequency, alert thresholds, incident pathways, rollback criteria, and rules for revalidation and regulatory change assessment.	Algorithmovigilance plan, version-specific monitoring reports, corrective and preventive action records, and evidence supporting each subsequent release [22]

## Algorithmovigilance Connects Design to Operation

Algorithmovigilance has been defined as organized methods for monitoring AI-driven health care for effectiveness and equity [22]. It extends beyond technical drift detection to include safety events, subgroup performance, user behavior, workflow consequences, complaints, and unintended access or exclusion. The term is established; the contribution of this viewpoint is to connect it to the same predeployment controls used for module boundaries, data permissions, federation logs, and cross-border decision rights.

A monitoring plan should be written before deployment and identify baseline performance, required data feeds, alert thresholds, review frequency, responsible persons, escalation routes, corrective and preventive actions, and criteria for rollback or retirement. This life cycle logic is consistent with the proposed good digital medicine practices (GDMP), a reference framework rather than a regulatory standard, which links continuous validation and real-world performance feedback to transparent, risk-proportionate change management [27]. A change that appears minor in code may alter intended purpose, clinical risk, consent expectations, or site comparability. Linking monitoring signals to a documented change-control pathway is therefore the operational bridge between compliance-by-design and life cycle governance.

## What Is INTERCEPT-Specific and What May Transfer

INTERCEPT-specific features include the focus on preclinical Crohn disease, blood biomarkers, people at increased risk, a multinational research consortium, and the possibility of a future prevention-oriented pathway. The eventual regulatory status of each component will depend on claims and implementation choices that remain under development.

More transferable artifacts are the function-and-boundary map, responsibility matrix, evidence matrix, permissions ledger, federated query log, model and data version record, release checklist, and algorithmovigilance plan. These artifacts can be adapted in other domains, but the 5 controls are not a universal architecture. Some projects may lawfully centralize data, some software will not be a medical device, some AI systems will not meet the AI Act’s high-risk criteria, and resource-constrained sites may need different technical controls. Transferability should be evaluated rather than assumed. At the cross-border level, the common-core and local-overlay approach is compatible with the GDMP convergence principle: shared operational expectations without assuming regulatory uniformity [27].

## Limitations

This viewpoint has important limitations. It reflects the interpretation of authors involved in one consortium, one disease area, and an early planning phase. The source selection was targeted rather than systematic, and no formal stakeholder study or consensus process was conducted. The framework may therefore reflect selection and interpretation bias. It has not been compared with alternative governance models, tested prospectively, or evaluated for time, cost, workload, participant experience, regulatory findings, clinical outcomes, or market access.

No AI model, federated production environment, consent platform, or clinical workflow is evaluated here. The national pathway examples are illustrative and may change. European rules and guidance continue to evolve, and their application depends on facts that cannot be resolved at the level of a general viewpoint. Disease-, product-, institution-, and jurisdiction-specific constraints may limit transfer. The framework should therefore be treated as a falsifiable planning hypothesis and not as proof of safety, effectiveness, scalability, or legal conformity.

## Conclusions

The central claim of this viewpoint is narrow: regulatory questions can be translated into design controls early enough to shape an AI-enabled digital medicine platform. The proposed contribution is to separate platform governance from module-level conformity, organize overlapping obligations into 5 linked controls, and connect predeployment traceability to postdeployment algorithmovigilance through a concrete life cycle example.

INTERCEPT provides a useful setting for this reasoning because its biomarker, data, AI, and cross-border components are still being defined. It does not yet demonstrate that compliance-by-design improves trust, clinical benefit, scalability, sustainability, or market access. Those propositions require prospective and comparative evaluation. Future studies should measure scientific validity, safety, participant and clinician experience, governance workload, time to regulatory decisions, nonconformities, implementation costs, and the consequences of change-control decisions. The value of the framework will depend on evidence that it improves decisions and outcomes, not on the completeness of its documentation alone.

## Abbreviations

CTR: Clinical Trials Regulation
DECIDE-AI: Developmental and Exploratory Clinical Investigation of Decision-support systems driven by Artificial Intelligence
EHDS: European Health Data Space
FUTURE-AI: Fairness, Universality, Traceability, Usability, Robustness, and Explainability in Artificial Intelligence
GDMP: good digital medicine practices
GDPR: General Data Protection Regulation
HTA: health technology assessment
IVDR: In Vitro Diagnostic Medical Devices Regulation
MDR: Medical Device Regulation
PROBAST+AI: Prediction Model Risk of Bias Assessment Tool for AI
TRIPOD+AI: Transparent Reporting of a Multivariable Prediction Model for Individual Prognosis or Diagnosis–AI
